# Somatosensory Influence on Platform-Induced Translational Vestibulo-Ocular Reflex in Vertical Direction in Humans

**DOI:** 10.3389/fneur.2020.00332

**Published:** 2020-05-14

**Authors:** Dieter F. Kutz, Florian P. Kolb, Stefan Glasauer, Hans Straka

**Affiliations:** ^1^Institute of Human Movement Sciences, Faculty of Behavioral and Social Sciences, Chemnitz University of Technology, Chemnitz, Germany; ^2^Department of Physiological Genomics, Institute of Physiology, Ludwig-Maximilian-University Munich, Munich, Germany; ^3^Computational Neuroscience, Institute of Medical Technology, Brandenburg University of Technology Cottbus-Senftenberg, Cottbus, Germany; ^4^Department of Biology II, Ludwig-Maximilians-University Munich, Munich, Germany

**Keywords:** locomotion, moving platform, retinal slip, bob, heave

## Abstract

The vestibulo-ocular reflex (VOR) consists of two components, the rotational VOR (rVOR) elicited by semicircular canal signals and the translational VOR (tVOR) elicited by otolith signals. Given the relevant role of the vertical tVOR in human walking, this study aimed at measuring the time delay of eye movements in relation to whole-body vertical translations in natural standing position. Twenty (13 females and 7 males) healthy, young subjects (mean 25 years) stood upright on a motor-driven platform and were exposed to sinusoidal movements while fixating a LED, positioned at a distance of 50 cm in front of the eyes. The platform motion induced a vertical translation of 2.6 cm that provoked counteracting eye movements similar to self-paced walking. The time differences between platform and eye movements indicated that the subject's timing of the extraocular motor reaction depended on stimulus frequency and number of repetitions. At low stimulus frequencies (<0.8 Hz) and small numbers of repetitions (<3), eye movements were phase advanced or in synchrony with platform movements. At higher stimulus frequencies or continuous stimulation, eye movements were phase lagged by ~40 ms. Interestingly, the timing of eye movements depended on the initial platform inclination. Starting with both feet in dorsiflexion, eye movements preceded platform movements by 137 ms, whereas starting with both feet in plantar flexion eye movement precession was only 19 ms. This suggests a remarkable influence of foot proprioceptive signals on the timing of eye movements, indicating that the dynamics of the vertical tVOR is controlled by somatosensory signals.

## Introduction

Vision is the dominant sensory modality in primates and humans that allows precise mapping and actively exploring the environment. However, during bipedal locomotion, the world cyclically shifts in the vertical direction and would thus impair optical image exploration. During everyday activities, visuo-vestibular reflexes induce counteracting eye/head movements that stabilize gaze on particular objects of interest (1). The VOR is the dominant motor reaction that provides fast compensation of retinal image motion during both translational and rotational head/body movements. Both reflex components work in concert and ensure optimal image stabilization during passive and active motions (2).

The rotational VOR could completely compensate visual perturbations such that the entire visual scene is stabilized on the retina. In contrast, during translational motion, the respective VOR is unable to stabilize the overall retinal image but only the image in the fixation plane (1, 3). Objects in front of the fixation plane will virtually move in the opposite direction to the translation, whereas objects behind the fixation plane will move in the direction of the translation [e.g., Figure 2 in (1)]. Accordingly, images of near and distant objects cannot be simultaneously stabilized on the retina during head translations. One solution is that the brain deliberately chooses a set point that minimizes relative image motion of the object of interest with respect to the background (4–8). Thus, the brain must decide whether to stabilize a particular image of a near object on the fovea or to minimize image motion relative to the background. Apparently, the brain chooses a compromise set point for the gain of the translational VOR that is optimal for the overall visual performance during locomotion (9). Thus, the translational VOR decreases conjugate retinal image slip and minimizes binocular disparities during self-induced motion or passive displacements. These vestibular-driven eye movements, which are vital for visual acuity, complement and work synergistically with visuo-motor reflexes (e.g., ocular following reflex) and depend on a decoding of either optic flow patterns or depth and binocular disparity cues [for review see Angelaki (2)].

During locomotion, the erect, straight-legged gait of humans induces substantial head translations in the vertical plane as well as side-to-side head translations (8, 10). These head perturbations have a predominant frequency range of 0.5–5.0 Hz (11–13). The rotational VOR counteracts head motion by inducing image stabilizing eye rotations during locomotion with latencies as short as 14–18 ms (14, 15). In contrast, cortical visual processing is too slow to contribute to gaze stabilization during locomotion-induced head perturbations [latency <85 ms, Table 1 in (1)]. However, some cortical areas are involved in visuo-motor procession of egomotion that aim at guiding locomotion (16). In the event that these reflexes are functionally incomplete, the resulting retinal image slip activates visual tracking mechanisms that operate as closed-loop negative feedback systems to produce eye movements that reduce the retinal image slip (3). Studies of gaze stability with subjects walking on a treadmill report a large retinal image slip (7–14°/s) and oscillopsia—illusory motion of a near target (17, 18). This suggests that the translational VOR minimizes the relative retinal vertical image slip of far and near objects, which optimizes the ability of the visual system to use motion parallax as a cue for navigation (6, 19).

This study thus aimed at a systematical determination of the time delays of vertical eye movements during vertical whole-body translations in natural upright position. The employed frequency range complied with that of normal locomotion. To evaluate the influence of somatosensory signals on the timing, the foot position at start was systematically altered.

## Methods

### Subjects and Ethics Statement

The study was performed with the permission of the ethics committee of the Ludwig-Maximilians-University Munich (#354-06) and was carried out in accordance with *The Code of Ethics of the World Medical Association* (*Declaration of Helsinki*). Twenty (13 females and 7 males) paid, healthy, young subjects [age 25.2 ± 0.6 years, mean ± standard error of mean (SEM)] participated in the study after having given written informed consent. Anthropomorphic characteristics of all participants are listed in Table 1. As a particularly important parameter, the length of the head–neck segment was calculated as the difference of the height of the eyes and the cervical vertebra C7. The mass of the head–neck segment was calculated by the formula given by (20) and (21):

(1) $$\begin{array}{l} head-neck\;mass=total\;body-weight*factor \end{array}$$

$$\begin{array}{l} with\;factor=\;\{\begin{array}{c} 0.08\;for\;women\\ 0.0826\;for\;men \end{array}\; \end{array}$$

**Table 1:** Mean values and standard deviations (sd) of anthropometric parameters of all subjects (*N* = 20).

	**Weight (kg)**	**Height above ground (cm)**						**Head–neck segment**	
		**Total**	**Eye**	**C7**	**Hip**	**Knee**	**Ankle**	**Length (cm)**	**Mass (kg)**
Mean	67.4	170.4	160.0	145.0	96.9	47.8	8.0	25.5	5.5
sd	11.0	9.2	9.0	7.9	5.8	4.7	0.8	2.7	0.9
Range	53–92	158–188	147–178	134–161	88–111	41–58	6–9	21–30	4.4–7.6

*The mass of the head–neck segment was calculated following Equation 1 [for references, see (20, 21)]*.

### Platform and Video-Oculography

**Figure 1:**
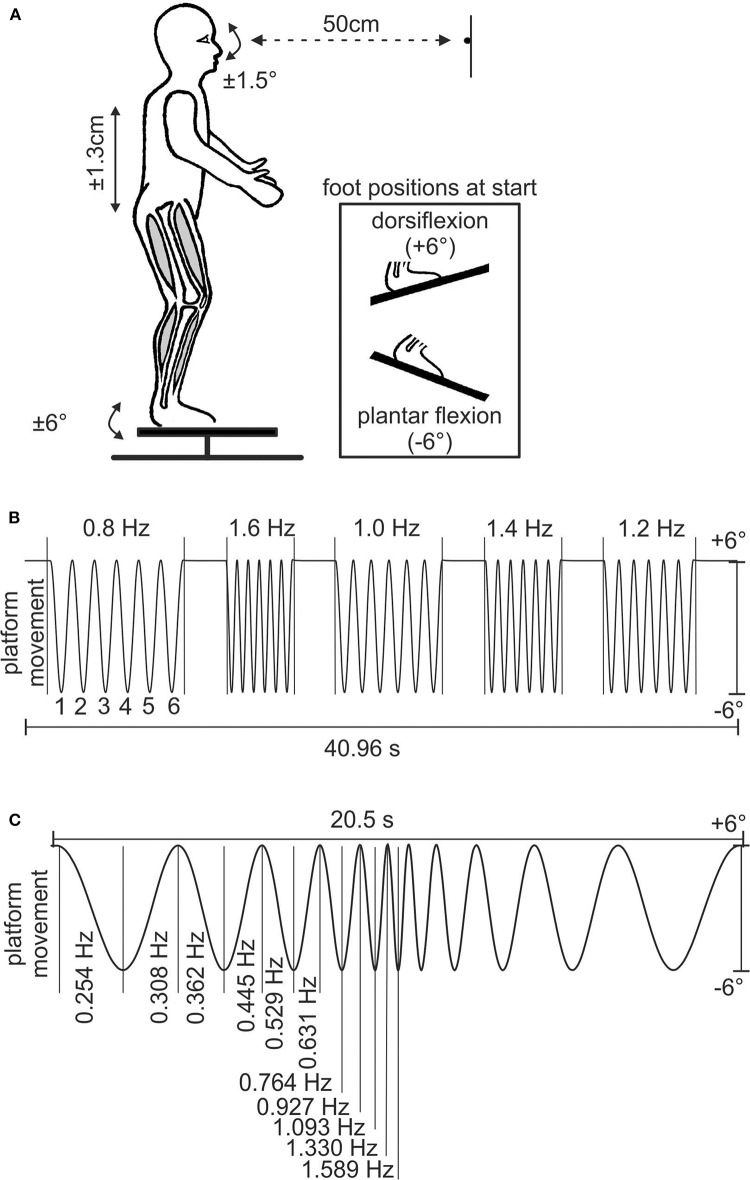
Experimental setup and stimulus paradigms. **(A)** Schematic of human subject on the motion platform with the LED positioned at the level of the eyes; the inset shows the different foot positions at the start of experiment B. **(B)** Stimulus sequence of experiment A; *y*-axis indicates ± 6° of platform movement, which resulted in a vertical translation of the head of ± 1.3 cm. Owing to the proximity of the LED, an eye movement amplitude of ± 1.5° is required for complete compensation of the head translation. The numbers above and below the sinusoids indicate stimulus frequency and number of stimulus cycle, respectively. **(C)** Stimulus sequence of experiment B depicting the 11 cycles of ascending–descending stimulus frequency profile of the platform motion; *y*-axis is the same as in B.

Subjects were asked to stand barefoot on a computer-controlled, motor-driven platform with a separate unit for each leg (Stopper, Burladingen, Germany), which was described elsewhere (22). Subjects were instructed to position each foot at the end of each half-platform with a distance of the ankles ~12.5 cm from the axis of the platform and to stand motionless on the platform (Figure 1A). The resultant distance between the heel centers was 23 cm, with the feet forming a slight V-shape. Platform movements were digitally, controlled, and recorded at 200 Hz/channel using a 12-bit A/D recording system (Micro Link 1000, WES, Germany) using custom-made software.

Eye movements were recorded with a mobile video-oculography system (EyeSeeCam, EyeSeeTec GmbH, Munich, Germany) at a sampling rate of 220 Hz. The spatial resolution of the eye-tracking device was 0.02° and the precision (relative error) on the order of 0.1° (23). Platform movements were synchronized to the start of the video-oculography by means of a specialized piece of equipment (Infrared SYNC Receiver for EyeSeeCam, EyeSeeTec GmbH, Munich, Germany).

### Experimental Procedure and Analysis

Subjects stood motionless at the rear end of the platform under room illumination with the instruction to fixate a LED at 50-cm distance (Figure 1A). The LED was positioned at the level of the eyes at horizontal platform position (i.e., 0°) at the midline of the subjects. All test persons confirmed to see the LED. Some of them wore contact lenses. Nevertheless, visual acuity was not specifically measured. Subjects were exposed to two different stimulus conditions for vertical whole-body movements (experiments A and B in natural upright position). To achieve this goal, neither the head was fixed relative to the body nor body segments were fixed to each other. The movement amplitude of the platform was ± 6° with respect to the horizontal plane and resulted in a vertical translation of ± 1.3 cm of the subject's body. This is similar to the vertical translation during self-paced walking (24, 25). To maintain fixation, subjects had to compensate the vertical translation by oppositely directed vertical eye movements. Owing to the proximity of the LED, the amplitude of the eye movements for complete compensation of the head translation must be ± 1.5° with respect to the straight-ahead position.

In experiment A, subjects were exposed to a stimulus consisting of five different frequencies (0.8, 1.6, 1.0, 1.4, and 1.2 Hz; see Figure 1B). The sequence consisted of six cycles at each frequency, separated by a 2-s interval of steady position. The total duration of a single trial was 40.96 s (Figure 1B). Subjects always started with feet in dorsiflexion (Figure 1A). Accordingly, the platform starting position was +6°. The sequence was presented to the subjects once for familiarization followed by five successive repetitions that were used for the analysis.

In experiment B, subjects were exposed to a stimulus of 22 up and down movements of the platform with logarithmically changing frequencies (26) at each change of movement direction (Figure 1C). Each trial was divided into two parts with the first half consisting of continuously increasing frequencies (0.254, 0.308, 0.362, 0.445, 0.529, 0.631, 0.764, 0.927, 1.093, 1.330, and 1.589 Hz) and the second half in reverse order. Initially, the sequence was presented to the subjects once with feet in plantar flexion for familiarization followed by five successive repetitions that were used for the analysis. For this purpose, the platform starting position was −6°. Immediately afterwards, the experiment was repeated starting with feet in dorsiflexion (Figure 1A), and five trials were recorded.

After the experiments, individual stride frequency during self-paced walking was determined to measure the time required to walk through an indoor corridor. The track was about 27 m long and was passed twice to count at least 40 strides.

### Data and Statistical Analysis

Data and statistical analysis were carried out using the language for statistical computing “R” (27). Vertical eye movements were down sampled to 200 Hz. Blinks were removed and substituted in three steps. First, all intervals with eye velocities exceeding 25°/s were detected. Second, start and end of such an interval were defined by a preceding and following value of velocity below 10°/s. The coherence between vertical eye movements and platform movements was analyzed using the R-package “WaveletComp, version 1.1” (28). For coherency analyses, the platform data were inverted such that a coherency of +1 indicates a phase of 0° and −1 a phase of 180°. Examples of coherence analyses are provided in the (Supplement Figures S1, S2). For significant coherence (*p* < 0.01, Bonferroni corrected), time differences between eye and platform movement were taken into account as phase. For the same interval, the gain was calculated as the amplitude of the eye movement divided by the amplitude of the theoretically necessary eye movement to compensate the platform movement. The vertical head angle in the pitch plane was calculated by integration of the head velocity obtained by the video-oculography system. The calculations of phase and gain were performed in experiment A separately for each of the six cycles of the five stimulus frequencies. For experiment B, the calculations were done separately for each half cycle of stimulation frequency, half-trial (increasing frequency and decreasing frequency), and foot position at start (dorsiflexion and plantar flexion). Differences between stimulus frequency and cycle in experiment A and between stimulus frequency, half-trial, and foot position at start in experiment B were analyzed by ANOVA with mixed design and multiple repetitions using the R-package “ez,” version 4.4-0 (29). Effect size $\eta_{\text{G}}^{2}$ is given to provide comparability (30). *p*-values were corrected following Greenhouse–Geisser, where appropriate.

## Results

A total of 20 subjects, aged 25.2 ± 0.6 years (mean ± SEM, 13 females), participated in both sets of experiments. For the experiments, participants were asked to stand on a motion platform that allowed applying vertical body translations. During the experiments, subjects had to fixate a white LED (Figure 1A), which, during vertical translations, provoked counteracting eye movements to maintain gaze on the target. In experiment A, five sequences of distinct platform motion frequencies between 0.8 and 1.6 Hz were tested (see Figure 1B). Experiment B consisted of sinusoidal platform motion with logarithmically ascending–descending stimulus frequency profile that ranged from 0.25 to 1.59 Hz (Figure 1C).

### Experiment A

**Figure 2:**
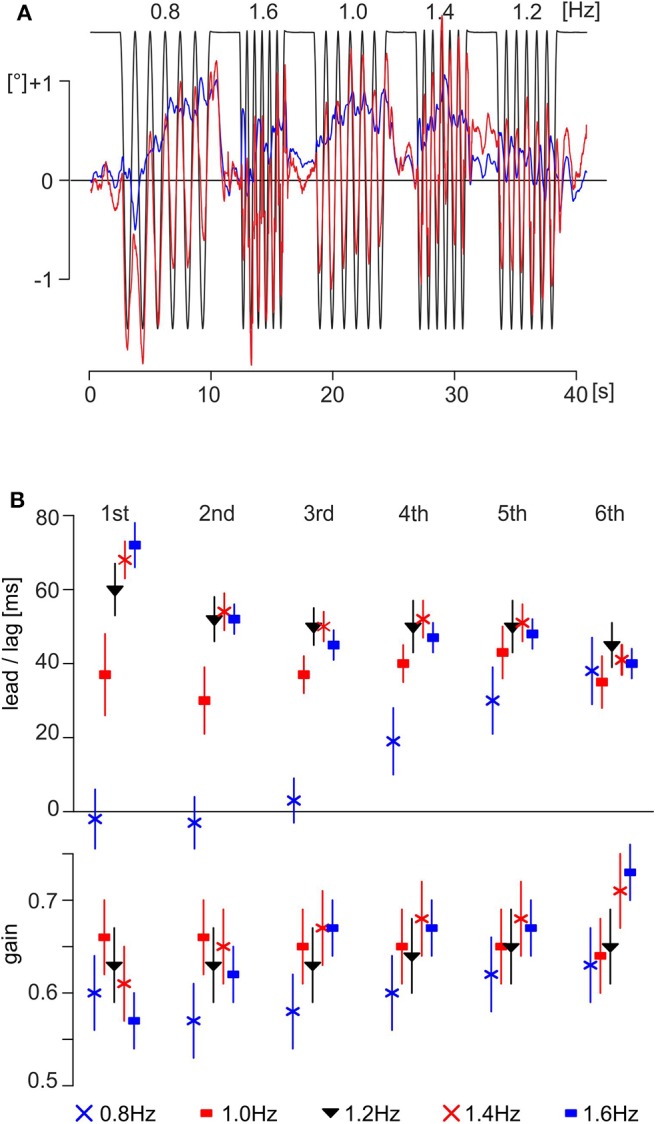
Data and results of experiment A. **(A)** Exemplary traces of one subject (male, 26.1 years); the eye motion is shown as a thick red line and the head angle in the pitch plane as a thin blue line. The theoretically necessary eye movement to fully compensate for the platform movement is shown as a thin black line. Note that the platform motion is shown inverted, scaled to overlap with eye position when the movement is compensatory. **(B)** Time delays (upper part) and gain (lower part) of evoked eye movements of all subjects (mean ± SEM; *N* = 20) in experiment A with respect to stimulus cycle.

In general, subjects were able to compensate the cyclic vertical body translations by dynamically adequate counteracting eye movements, however, with two limitations: first, eye movement magnitudes were smaller than required for complete motion compensation (Figure 2A; note that platform movement is inverted); second, the eye position in the orbital cavity was not stable but oscillated around the horizontal straight ahead position (0° in Figure 2A). Interestingly, the head angle in the pitch plane describes an envelope of the eye movement maxima (Figure 2A, blue and red lines), with a tendency of the head pointing upwards. The original position is reached again and again during the stimulation pauses. This is an indication that the participant is changing his posture slightly by lowering the body. It seems that the participant tries to attenuate the stimulation by allowing relative movements of joints (e.g., vertebrae, hip, knee, and ankle). This changed posture is compensated by an inclination in the pitch plane. During the pauses, he or she stretches again and the pitch angle returns to zero.

The time delays of the eye movements with respect to the vertical body motion differed depending on stimulus frequency and cycle number (Figure 2B, upper part). The fastest reactive eye movements were observed for the first and second cycles of the motion sequence at 0.8 Hz with a lag of 2 ± 6 ms (mean ± SEM) and a lead of −4 ± 5 ms, respectively (Figure 2B, upper part, blue X at the first and second cycles). Interestingly, the time delays at 0.8 Hz increased over the following four cycles to reach a steady state of 37 ± 7 ms for the sixth cycle (Figure 2B, upper part, blue X at sixth cycle). The longest latency of 72 ± 4 ms was observed for the first cycle of the motion sequence at 1.6 Hz (Figure 2B, upper part, filled blue squares at the first cycle) that, however, decreased during the following five cycles to 40 ± 2 ms for the sixth cycle (Figure 2B, upper part, filled blue squares at the sixth cycle). A similar behavior was encountered for the sequences with vertical motion stimulus frequencies of 1.2 and 1.4 Hz. At both stimulus frequencies, the time delays decreased from the first to sixth cycle (Figure 2B, upper part, filled black triangles and red X for 1.2 and 1.4 Hz, respectively). Interestingly, however, during stimulation with a motion frequency of 1.0 Hz, the time delays remained relatively constant over all cycles with a mean of 37 ± 2 ms (Figure 2B, upper part, filled red squares). This suggests that platform motion at 1.0 Hz appears to resonate with a particular intrinsic temporal feature at variance with higher or lower stimulus frequencies. ANOVA with mixed design and multiple repetitions confirmed the significant effects (α = 0.001, Bonferroni corrected) for the main effect *frequency* and for the interaction *frequency* × *number of cycle* (Table 2). The gain was similar for all frequencies and cycles (Figure 2B, lower part), with a range from 0.57 to 0.73 (first and sixth cycles for a stimulation at 1.6 Hz, filled blue squares in Figure 2B, lower part) and a total mean value of 0.64. ANOVA with mixed design and multiple repetitions showed a significant effect only for the interaction *frequency* × *number of cycle* (α = 0.001, Bonferroni corrected, Table 2). This interaction is mainly driven by the increasing gain for stimulation frequencies 1.4 and 1.6 Hz during repetitions (Figure 2B, lower part, red X and filled blue squares, respectively).

**Table 2:** Statistical parameters of the ANOVA of experiment A.

**Experiment A**	**Effect**	**Degree of freedom (nominator)**	**Degree of freedom (denominator)**	***F***	***P***	**$\eta_{\text{G}}^{2}$**
Timing	Frequency	4	76	22.10	**5.12** * **10**^**−8**^	0.21
	Cycle	5	95	4.21	2.68 * 10^−2^	0.02
	Frequency × cycle	20	380	10.78	**1.09** * **10**^**−5**^	0.09
Gain	Frequency	4	76	1.92	1.64 * 10^−1^	0.02
	Cycle	5	95	7.61	5.02 * 10^−3^	0.01
	Frequency × cycle	20	380	6.75	**1.15** * **10**^**−5**^	0.02

*p-values were corrected following Greenhouse–Geisser, where appropriate. Significant p-values after Bonferroni correction at a level of α = 0.001 are indicated in bold*.

**Figure 3:**
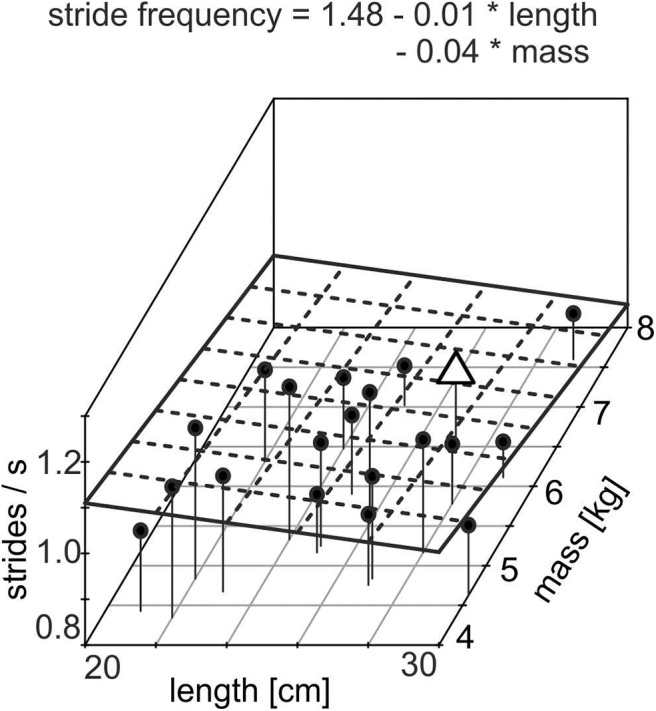
Three-dimensional scatterplot of individual stride frequency with respect to length and mass of the individual head–neck segment of all subjects (*N* = 20); individual values are shown as black dots; the plane given by the dashed lines shows the regression plane given by the equation. The data of the subject, shown in Figures 2A, 4A, are indicated by an open triangle. Note the significant decrease in stride frequency with increasing length and mass (adjusted *R*^2^ = 0.369).

In order to allow a comparison of vertical eye motion dynamics during passive perturbations with those during locomotion, we determined individual stride parameters during self-paced walking for each subject. The mean stride frequency was 0.99 ± 0.02 strides/s (mean ± SEM), the mean length of the head–neck segment was 25.5 ± 2.7 cm, and the mean mass of the head–neck segment was 5.5 ± 0.9 kg (Table 1). Interestingly, stride frequency decreases significantly with increasing length of the head–neck segment [*F*_(1, 12)_ = 7.17, *p* = 0.020, $\eta_{\text{G}}^{2}$ = 0.374] and with increasing mass [*F*_(1, 12)_ = 5.53, *p* = 0.037, $\eta_{\text{G}}^{2}$ = 0.316] (Figure 3, adj. *R*^2^= 0.369). Assuming that the head–neck segment is an inverted version of a simple gravity pendulum with a pendulum length of 25.5 cm, the swing period approximates to 1.01 s, which corresponds to stride frequency. Hence, subjects prefer a stride frequency that matches the physical characteristics of their head–neck system. These results potentially explain the phase advance of counteracting eye movements during vertical motion platform oscillations when the stimulus frequency is lower than the individual stride frequency. This assumption was further tested in experiment B, where a stimulus sequence with lower frequencies was used.

### Experiment B

**Figure 4:**
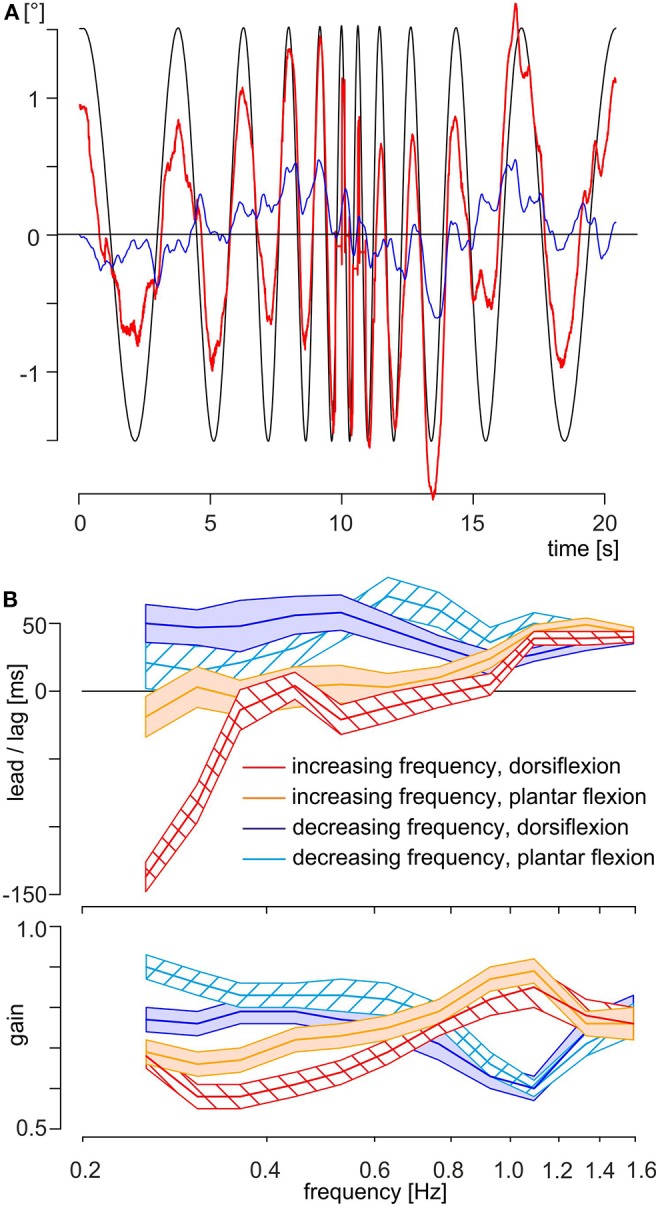
Data and results of experiment B. **(A)** Exemplary traces of the same subject as shown in Figure 2; the eye motion is indicated as a thick red line and the head angle in the pitch plane as a thin blue line. The theoretically necessary eye movement to fully compensate for the platform movement is shown as a thin black line. Note that the platform motion is shown inverted, scaled to overlap with eye position when the movement is compensatory. **(B)** Time delays (upper part) and gain (lower part) of evoked eye movements of all subjects (*N* = 20) in experiment B with respect to stimulus frequency and separated by half-trial and position of the feet at start. The mean is shown as a colored thick line and SEM as a colored area with, red line and red left oblique hatched area: increasing frequency with feet in dorsiflexion, orange line and orange filled area: increasing frequency with feet in plantar flexion, blue line and blue filled area: decreasing frequency with feet in dorsiflexion, cyan line and cyan right oblique hatched area: decreasing frequency with feet in plantar flexion.

Application of sinusoidal platform motion with a logarithmically ascending–descending stimulus frequency profile (Figures 1C, 4A) provoked counteracting eye movements with corresponding dynamics. As in experiment A, the eye motion did not completely offset the vertical head/body translation, neither at low nor at higher stimulus frequencies (Figure 4A). During the initial part of the stimulus, subjects often anticipated or phase-timed the eye movements (see interval of 0–5 s in Figure 4A). In contrast, eye movements during later stimulus cycles were often phase lagged with respect to the vertical head/body perturbation (see interval of 12–17 s in Figure 4A). The head angle in the pitch plane rises slowly until 9.2 s, then tilts slowly until 13.4 s, and finally rises again (Figure 4A, blue line). A correlation between head position and eye position is not visible (Figure 4A, blue line and red line). Again, the participant tries to attenuate the stimulation by a slight change in his posture, which resulted by allowing relative movements of corresponding joints. As this stimulation is continuous and there is no pause time, the inclination of the head reaches a value that is perceived as to great and a counteracting movement of the head is initiated (e.g., Figure 4A, blue line approximately at 9.2 s). Overall, subjects appeared to be unable to predict the forthcoming stimulus cycle on the basis of the prior cycle.

In general, the time delays differed owing to the continuously increasing or decreasing stimulus frequencies and owing to the foot position at start (Figure 4B, upper part). During increasing frequencies up to 0.76 Hz, eye movements were phase leading or in phase with the platform movements. Note that during this interval the mean time delay of the response with feet starting in dorsiflexion was significantly shorter than the corresponding time delay with feet starting in plantar flexion (Figure 4B, upper part, red and orange thick lines). At higher stimulus frequencies, that is, from 0.97 to 1.59 Hz, eye movements were phase lagged relative to the platform movements and independent of foot position, with a mean lag of 40 ± 2 ms (mean ± SEM) at 1.59 Hz. During the subsequent stimulus sequence with decreasing frequencies, eye movements remained phase lagging independent of stimulus frequency and foot position (Figure 4B, upper part, blue and cyan thick lines for dorsiflexion and plantar flexion, respectively) with a mean time delay of 41 ± 11 ms. ANOVA with mixed design and multiple repetitions confirmed the significance (α = 0.001, Bonferroni corrected) for the main effects *frequency* and *half-trial*, and for the interaction *frequency* × *half-trial, frequency* × *foot position at start*, half-*trial* × *foot position at start*, and *frequency* × *half-trial* × *foot position at start*. The significance of the main effect *foot position at start* reached after Bonferroni correction only the value of a trend (Table 3). The gain was similar for all frequencies and position of the feet (Figure 4B, lower part) with a range from 0.58 (increasing frequencies, dorsiflexion at 0.362 Hz) to 0.89 (increasing frequencies, plantar flexion at 1.093 Hz) and a total mean value of 0.74. ANOVA with mixed design and multiple repetitions showed a significant effect only for the interaction *frequency* × *half-trial* (α = 0.001, Bonferroni corrected, Table 3). This interaction is mainly driven by the higher gain for the stimulus sequence with decreasing frequencies below 0.764 Hz (Figure 4B, lower part, red and orange thick lines vs. blue and cyan thick lines).

**Table 3:** Statistical parameters of the ANOVA of experiment B.

**Experiment B**	**Effect**	**Degree of freedom (nominator)**	**Degree of freedom (denominator)**	***F***	***p***	**$\eta_{\text{G}}^{\text{2}}$**
Timing	Frequency	10	190	12.78	**1.79** * **10**^**−5**^	0.12
	Half-trial	1	19	52.59	**6.99** * **10**^**−7**^	0.14
	Foot position at start	1	19	7.99	1.08 * 10^−2^	0.02
	Frequency × half-trial	10	190	21.27	**9.26** * **10**^**−10**^	0.11
	Frequency × foot position at start	10	190	4.58	**2.86** * **10**^**−3**^	0.02
	Half-trial × foot position at start	1	19	22.78	**1.32** * **10**^**−4**^	0.02
	Frequency × half-trial × foot position at start	10	190	14.81	**2.76** * **10**^**−7**^	0.06
Gain	Frequency	10	190	0.75	0.48	0.01
	Half-trial	1	19	7.74	1.19 * 10^−2^	0.01
	Foot position at start	1	19	6.98	1.61 * 10^−2^	0.03
	Frequency × half-trial	10	190	55.18	**2.98** * **10**^**−15**^	0.21
	Frequency × foot position at start	10	190	3.40	2.63 * 10^−2^	0.02
	Half-trial × foot position at start	1	19	0.10	0.76	0.00
	Frequency × half-trial × foot position at start	10	190	2.43	7.44 * 10^−2^	0.01

*p-values were corrected following Greenhouse–Geisser, where appropriate. Significant p-values after Bonferroni correction at a level of α = 0.001 are indicated in bold. For the effect “half-trial,” the frequency increased continuously during the first half and decreased continuously during the second half. The foot position at start was dorsiflexion or plantar flexion*.

## Discussion

This study aimed at determining the time delays of counteracting vertical eye movements induced during cyclic vertical body movements. Eye movements preceded body movements during the first cycle of stimulus frequencies <1 Hz but lagged body motion at stimulus frequencies > 1 Hz. During continuous stimulation, the time delays of eye movements reached a steady-state value of ~40 ms. For frequencies between 1.9 and 2.3 Hz, latencies from 20 ms (6, 31) up to 50 ms (32) and 70 ms (33) were reported. For stimulation frequencies below 1 Hz, lagging behavior was reported [38 ms at 0.73 Hz, (33)]. In this case, the reported value was calculated over a period of more than 20 s during stimulation with a sum-of-sines waveform. Hence, the reported value refers to steady state and is similar to our value. Comparable observations were also reported for horizontal translational motion stimulation on a sled. Phase-advanced eye movements with 111 and 56 ms were reported for stimulation frequencies of 0.25 and 0.5 Hz, respectively (34), whereas eye movements lagged body translation for 8 ms at a stimulus frequency of 0.5 Hz (35). Independent of stimulus frequency, the gain of the eye movements was lower than necessary for compensation with a total mean value of 0.64 and 0.74 for experiments A and B, respectively. This is consistent with the reported gain spectrum (0.42–0.75) of earlier reports (6, 31–33). Interestingly, the mean time lag of 40 ms in the current study is maintained during an experimental sequence even when stimulus frequency decreased below 1 Hz. This suggests that this magnitude is the preferred frequency for this reactive motor behavior, which perfectly matches the value (mean: 0.99 strides/s) for self-paced stride frequency. Moreover, this coincidence extends to normal walking at a speed of 4–5 km/h, equivalent to a stride frequency of ~1 stride/s in normal subjects at which the energy costs are the best compromise between movement effort and muscle efficiency (25).

During vertical body movements at a frequency <1 Hz, the time delays of the eye movements depended on the starting position. For the first two stimulation frequencies, eye movements started earlier when feet at start were in dorsiflexion than in plantar flexion (Figure 4B, upper part, red and orange thick lines). This might derive from the fact that during vertical body movements, the LED elicits an optokinetic reaction or pursuit eye movement. This assumption complies with a larger influence of vision up to stimulus frequencies of 0.4 Hz as the response gains are close to 1, whereas gains decrease significantly at higher visual motion stimulus frequencies (36–40). Different results have been reported regarding the directional symmetry of the gain. Symmetrical responses were reported on a group level by (36) and (40), whereas individual subjects could either have higher gains for downward directed (39, 40) or upward movements (36). The later behavior was also described by (39). A prevalence of the upward direction on group level was reported by (37) and by (38). It must be noted, however, that in our experiment the platform direction and, hence, the stimulus direction reverses after every half wave and, second, that the direction of the visual field motion depends on the point of fixation (1, 3). Assuming that the direction of stimulation of the visual field behind the LED would have a stronger influence on the time delays, the results must be reordered. The sequence for upward direction would be 0.254 Hz/dorsiflexion, 0.308 Hz/plantar flexion, 0.362 Hz/dorsiflexion, and so forth, and for the downward direction 0.254 Hz/plantar flexion, 0.308 Hz/dorsiflexion, 0.362 Hz/plantar flexion, and so forth (Supplementary Table S1). ANOVA with mixed design and multiple repetitions showed no significant result for the main effect *direction* and the interaction *direction* × *half-trial*. The only significant effect was for *half-trial* demonstrating that the system reached a steady state during the first half of the trial (Figure 4B, upper part). Hence, the differences in time delays with respect to the starting position of the feet cannot be exclusively explained by visuo-motor contributions.

At the start, the test person is in an upright position in both forms of stimulation (dorsiflexion and plantar flexion). Therefore, the signals of the otolith system are comparable. It can be assumed that the test person tries to maintain the original posture and orientation during the stimulation. Therefore, he or she is dependent on somato-sensory signals as a replacement for the otolith system. This is important because it indicates the likelihood of proprioceptive influences on eye movement behavior. Mullick et al. (41) studied electromyographic activity of three lower limb muscles (*musculus gastrocnemius, m. soleus*, and *m. tibialis anterior*) in human standing quietly on a inclined surface similar to our experiments. They show that the *m. gastrocnemius* and the *m. soleus* are more active when standing on a descending platform (plantar flexion) compared to an ascending to ascending platform (dorsiflexion) whereas the *m. tibialis* anterior does not show significant differences (see Figure 2A of that paper). The latter one is co-activated with the former during posture stabilization after perturbation [e.g., Kolb et al., (42)]. The different muscle activation during the initial situations is accompanied by corresponding gamma-motoneuron activity, which in turn is associated with a different sensitivity of the muscle spindle. Because the *m. tibialis* does not contribute significantly in both initial situations, proprioceptive perception is driven by the different activation of the *m. gastrocnemius* and *m. soleus*. We are convinced that this influences extraocular motor function and thus the different response behaviors. Thus, this extraocular motor behavior can be appropriated to the responses of the ankle extensor.

In addition, recordings of the *m. gastrocnemius*-innervating tibial nerve [e.g., Jones and Small (43), Riffel and Stohr (44), and Tinazzi et al., (45)] indicated that the average latency from the ankle to the brainstem is ~29 ms, somewhat longer than the shortest latency for eye muscle activation in humans (11–16 ms) or following step-like head translation [16 ms; (5)]. A value of 10–15 ms therefore appears to be the typical delay for motion-induced eye movements as suggested from recordings of single motor unit activity of the *inferior oblique* and *inferior rectus* eye muscles following skull vibration and sound application in human subjects (46). Following arithmetic addition of the latencies from the ankle to the brainstem and from the brainstem to the eye muscles, it is likely that ankle movements could in fact evoke eye muscle activity within 40–46 ms.

Subjects apparently learn to counteract gaze changes induced by vertical body movements using sensory information from the feet and/or other parts of the leg. Altered gravity—by unloading body weight or by exposition to hypogravity and hypergravity during parabolic flights—is able to change head and eye movements (47–51). For example, head pitch amplitude increases ~1° with respect to normal walking when subjects perform a 30-min unloaded locomotor training while watching a movie on a computer screen as gaze task. During the post-training phase, subjects return to normal behavior after just four walking trials of 30 s each (51). In addition, upright-standing subjects during parabolic flights produce a sustained downbeat nystagmus during sudden hypogravity and a sustained upbeat nystagmus during sudden hypergravity (47–50). Recent studies have shown that a positional nystagmus is common in healthy subjects under normal gravity depending on the subject's position (52, 53) and can be explained by the effect of gravity on otolith pathways for static eye position (54). Following (50), the results of these different experiments lead to a set of important conclusions: “(1) sustained downbeat or upbeat nystagmus in altered gravity unmasks a fundamental, permanent influence of normal gravity on vertical eye position, which is maximal in the upright position and minimal in the horizontal position of the head; (2) consequently, to stabilize vertical eye position in erect positions of the head, a neuronal system is required to counteract gravity permanently.” Nevertheless, human subjects are able to adapt to prolonged changes in gravity (50, 51), suggesting that peripheral somatosensory signals converge with otolith signals. During unloaded locomotor training, the central nervous system must adapt motor behavior to reduced somatosensory signals from the foot plantar sole as well as from other parts of the body. Hence, a fast mechanism to readapt vestibular signals by somatosensory signals must therefore exist. A putative structure for such adaptive computations is the cerebellum (54–57). Thus, in conclusion, during walking, eye movements are coupled to the somatosensory system through a modulation of the vestibulo-ocular reflex.

## Data Availability Statement

The datasets generated for this study will not be made publicly available due to local law restrictions.

## Ethics Statement

The studies involving human participants were reviewed and approved by Ludwig-Maximilians-University Munich, Faculty of Medicine. The patients/participants provided their written informed consent to participate in this study.

## Author Contributions

DK and FK performed data collection and analysis. The manuscript was written in sections by the different authors and was compiled by DK. All authors read and approved the final manuscript and contributed to the study conception and design.

## Conflict of Interest

The authors declare that the research was conducted in the absence of any commercial or financial relationships that could be construed as a potential conflict of interest.
