# Supplementary material for: Somatosensory Influence on Platform-Induced Translational Vestibulo-Ocular Reflex in Vertical Direction in Humans
Source: Front Neurol. 2020 May 14;11:332. doi: 10.3389/fneur.2020.00332 (PMC7238866; doi:10.3389/fneur.2020.00332)
Supplement: Supplementary file 1 [file Data_Sheet_1.pdf]

Supplement Figure S1: Wavelet coherency values for the data shown in Fig. 2A. The coherency between vertical eye movements and platform movements were analysed using the R-package "WaveletComp, version 1.1" (Roesch & Schmidbauer, 2018). The time of stimulations is shown with black traces at the abscissa. The stimulation frequency is given as period at the ordinate. Coherency values are colour coded. The direction of the black arrows indicates whether eye movements lead (downward) or lag (upward) the plattform movement. Regions with significant coherency values are circumscribed by a white line. The five intervals with different stimulation frequencies are clear visible. Noteworthy, during stimulation with 0.8Hz and 1.0Hz eye movements are more or less in time whereas during the other frequencies they lag behind.

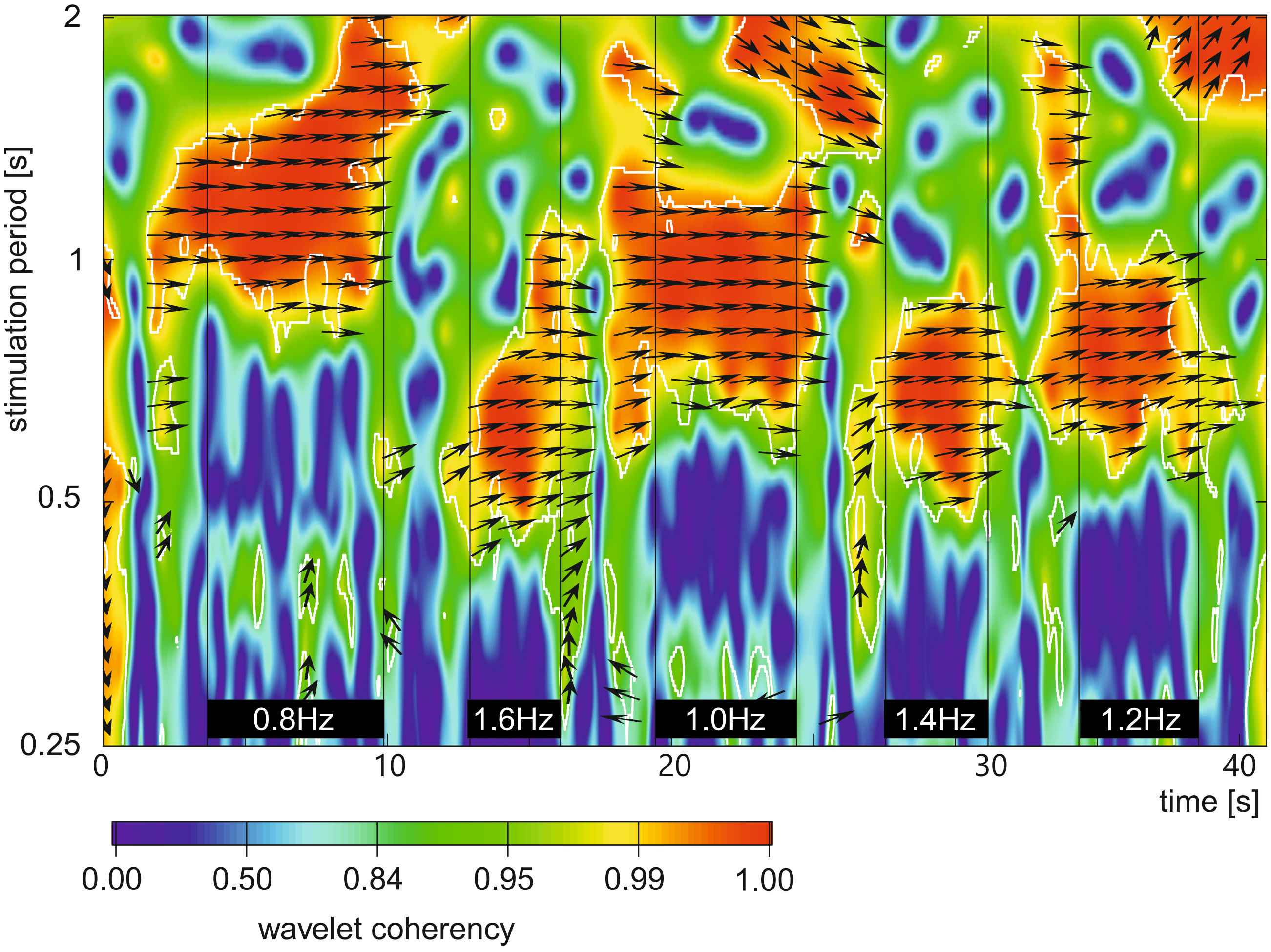

Supplement Figure S2: Wavelet coherency values for the data shown in Fig. 4A. Other details as in Fig. S1.
Noteworthy, during higher stimulation frequencies (stimulation period below 1s) eye movements lag platform movements behind (arrows point upwards).

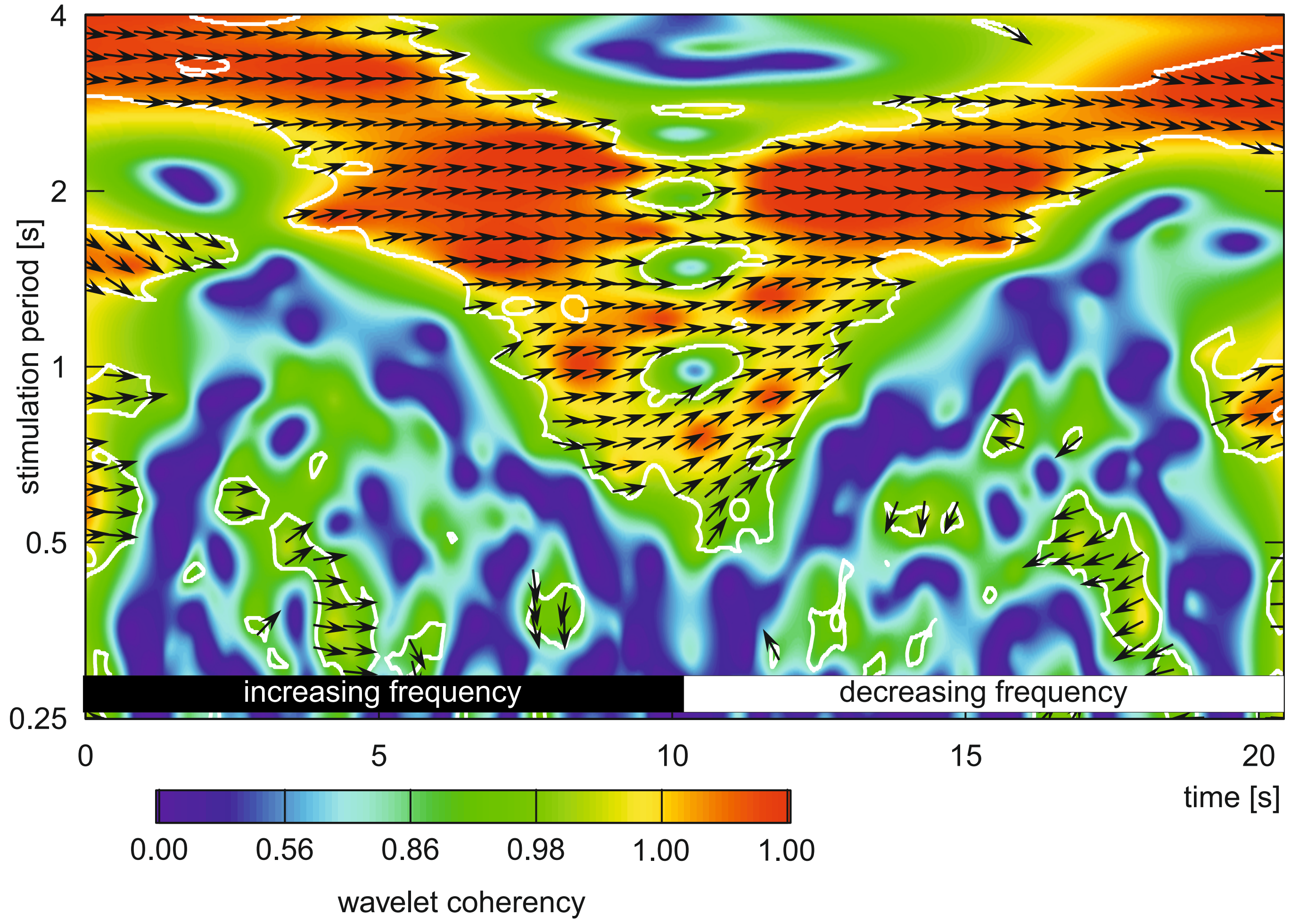

Supplementary Table S1 Mean latency (± SEM in ms) of eye movements with respect to stimulus frequency, trial-half (increasing frequency *versus* decreasing frequency) reordered by direction of visual stimulation (upward *versus* downward) in experiment B. Negative values indicate that eye movement precedes platform movement and positive value that eye movement lags platform movement.

	increasing frequency		decreasing frequency	
frequency [Hz]	upward	downward	upward	downward
0.254	-19 ± 15	-137 ± 11	21 ± 19	50 ± 14
0.308	-83 ± 14	3 ± 15	47 ± 13	15 ± 18
0.362	-5 ± 13	-14 ± 15	21 ± 13	48 ± 19
0.445	4 ± 10	3 ± 15	56 ± 14	32 ± 15
0.529	5 ± 14	-21 ± 10	49 ± 12	58 ± 13
0.631	-12 ± 11	3 ± 10	46 ± 11	70 ± 14
0.764	10 ± 8	-3 ± 9	60 ± 13	33 ± 8
0.927	5 ± 8	24 ± 7	21 ± 9	36 ± 11
1.093	44 ± 5	39 ± 5	50 ± 8	27 ± 5
1.330	39 ± 5	49 ± 5	36 ± 6	45 ± 6
1.589	43 ± 4	40 ± 4	43 ± 4	39 ± 4
